# Complex and Dynamic Effects of an Extreme Low Temperature Weather Event on Invasive Plant Populations and Resident Communities

**DOI:** 10.1111/gcb.70113

**Published:** 2025-03-03

**Authors:** Maurício Cruz Mantoani, Conor Sweeney, Bruce A. Osborne

**Affiliations:** ^1^ UCD School of Biology and Environmental Science University College Dublin (UCD) Dublin Ireland; ^2^ Institute of Astronomy, Geophysics and Atmospheric Science (IAG) University of São Paulo (USP) São Paulo Brazil; ^3^ UCD School of Mathematics and Statistics UCD Earth Institute Dublin Ireland; ^4^ UCD School of Agriculture and Food Science UCD Earth Institute Dublin Ireland

**Keywords:** biomass productivity, competition, ecosystem resilience, environmental thresholds, *Gunnera tinctoria*, invasive alien plants, competição, *Gunnera tinctoria*, limiares ambientais, plantas exóticas invasoras, produtividade de biomassa, resiliência do ecossistema

## Abstract

Whilst it is often assumed that invasive plant species may benefit more from climate change than native species, there is little empirical data on how they, and the communities they invade, respond to extreme weather events (EWEs). Here, we show that the effects of a low temperature EWE can result in a dramatic reduction in both vegetative and reproductive growth of invasive populations of *Gunnera tinctoria* , although a significant recovery was found within 1 year after its occurrence. Whilst the EWE decreased both the leaf/petiole numbers of mature plants, the major impact was on leaf expansion and a decrease in the size/number of inflorescences. Concomitant with the reduction in growth of *G. tinctoria* , there was a 5‐fold increase in the number of resident species emerging in invaded areas, which largely persisted after the recovery of the invasive populations. Although the growth of *G. tinctoria* seedlings was also reduced, this was relatively small, and growth and development resumed almost immediately after the EWE. In comparison, the resident plant community was largely unaffected by the EWE either due to the later initiation of growth and/or because of their greater resilience to episodic low temperature events. Our results show that an EWE of this magnitude can have complex time‐dependent effects on plant invasions and invaded communities, with a greater impact on the established invasive plants compared to newly recruited seedlings. Based on an assessment of historical climatic data, these long‐lived populations have been exposed to EWEs of a similar or greater magnitude in the past, which have not constrained their spread or compromised recruitment. Given the likelihood of an almost complete absence of low temperature EWEs of similar magnitude in the future, any temporary restrictions on the growth of invasive *G. tinctoria* and other similar invasive species populations are likely to be small.

## Graphical abstract

Invasive plant species might benefit more from climate change, though evidence for that is not clear. Here, we show that the effects of a low‐temperature extreme weather event can result in a dramatic reduction in both vegetative and reproductive growth of invasive populations of *Gunnera tinctoria* (giant rhubarb) in the west of Ireland. The extreme event had complex time‐dependent effects on plant invasions and invaded communities, but a significant recovery was found within 1 year after its occurrence. In the future, such extreme events are likely to be rare and thus restrictions on invasive plant populations will be small.

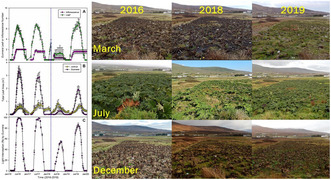

## RESUMO

Embora muitas vezes se suponha que as espécies de plantas invasoras possam se beneficiar mais das mudanças climáticas do que as espécies nativas, há poucos dados empíricos sobre como elas e as comunidades que elas invadem respondem a eventos climáticos extremos (ECEs). Aqui, mostramos que os efeitos de um ECE de baixa temperatura podem resultar em uma redução dramática no crescimento vegetativo e reprodutivo de populações invasoras de *Gunnera tinctoria*, embora uma recuperação significativa tenha sido encontrada dentro de um ano após sua ocorrência. Enquanto o ECE diminuiu o número de folhas/pecíolos das plantas maduras, o maior impacto foi na expansão foliar e na diminuição do tamanho/número de inflorescências. Concomitante à redução do crescimento de *G. tinctoria*, houve um aumento de 5 vezes no número de espécies residentes emergentes em áreas invadidas, que persistiram em grande parte após a recuperação das populações invasoras. Embora o crescimento das plântulas de *G. tinctoria* também tenha sido reduzido, este foi relativamente pequeno, e o crescimento e o desenvolvimento foram retomados quase imediatamente após o ECE. Em comparação, a comunidade de plantas residentes não foi afetada pelo ECE devido ao início tardio do crescimento e/ou por causa de sua maior resiliência a eventos episódicos de baixa temperatura. Nossos resultados mostram que um ECE dessa magnitude pode ter efeitos complexos e dependentes do tempo sobre os processos de invasão por plantas e as comunidades invadidas, com maior impacto nas plantas invasoras estabelecidas em comparação com mudas recém‐recrutadas. Com base em uma avaliação de dados climáticos históricos, essas populações foram expostas ao longo do tempo a ECEs de magnitude semelhante ou maior no passado, o que não restringiu sua disseminação nem comprometeu o recrutamento. Dada a probabilidade de uma ausência quase completa de ECEs de baixa temperatura de magnitude semelhante no futuro, quaisquer restrições temporárias ao crescimento de *G. tinctoria* e outras populações de plantas invasoras semelhantes provavelmente serão pequenas.

## 1 Introduction

It is often assumed that invasive plant species may benefit more from climate change than native species (Polgar and Primack 2011; Wolkovich and Cleland 2011; Wolkovich et al. 2013; Polgar et al. 2014; Ratcliffe et al. 2024), although experimental evidence for this is limited and there is little‐to‐no information on how they will respond to extreme weather events (EWE), many of which are projected to increase with global warming (Easterling et al. 2000; Allan and Soden 2008; Jentsch and Beierkuhnlein 2008). The early growth of many plant invaders (Polgar and Primack 2011; Wolkovich and Cleland 2011; Wolkovich et al. 2013; Polgar et al. 2014) may make them particularly sensitive to EWEs that occur early in the growing season, as they may be more susceptible to extreme temperatures and/or water availability during the early stages of growth (Laube et al. 2015; Vitasse et al. 2018) in comparison to later growing native species. Some of the common effects of climate change on invasive plants that have already been reported, including an advancement of leaf‐out in spring and shifts in flowering patterns (Polgar and Primack 2011; Wolkovich and Cleland 2011; Wolkovich et al. 2013; Polgar et al. 2014), as well as an extended leaf phenology and delayed abscission in autumn (Fridley 2012; Gallinat et al. 2015), could make them more susceptible to the effects of early or late occurring EWEs. As a consequence, contrary to generalized global warming projections indicating that invasive plants may benefit from climate change (Bradley et al. 2010; Fennell et al. 2013; Dainese et al. 2017), this could lead to a contraction rather than an expansion in the distribution of introduced plant species. However, this is likely to depend on the timing, frequency, duration, and severity of an EWE, and how quickly invasive plants and the communities they invade can recover afterwards (Diez et al. 2012).

Climate change can also impact on competitive interactions between invasive plants and native species, as well as their distribution and spread (Groeneveld et al. 2014). Whilst native plants may also respond to climate warming (Cook et al. 2012), plant invaders could benefit disproportionally more from climate change if this favors their potential for early growth (Polgar and Primack 2011; Wolkovich and Cleland 2011; Wolkovich et al. 2013; Polgar et al. 2014), as this would reduce any competition with native species during early establishment. Although early seedling growth could contribute to the success of at least some invasive species (Ni et al. 2018), there is limited information on developmental and phenological differences between mature plants and seedlings to aid our understanding of how significant this is for the invasion process and the spread of invasive plant populations. In terms of the occurrence of extreme low temperature events, climate change‐related advancement of the growing season could pose significant risks for plants that initiate growth earlier (Bradley et al. 2010; Laube et al. 2015). Since the date of first spring frost may not change significantly (Vitasse et al. 2018), growing earlier brings the risk of low temperature‐related impairment of growth and reproduction. Given that the growth period is strongly controlled by regional climate history, native species may display more conservative traits that have made them more resistant to EWEs and/or allowed them to evade most of their effects through adaptation to a shorter growing season (Zohner and Renner 2017).

Due to their stochastic nature, it is difficult to predict the timing of an EWE (Jentsch and Beierkuhnlein 2008) and there are few observational examples in the literature. The myriad of potential impacts associated with the effects of an EWE on native and introduced plants, besides changes in phenology (Jentsch et al. 2009; Reyer et al. 2013), includes shifts in community composition (Parmesan et al. 2000) and modifications in ecosystem processes, such as nutrient cycling (Jentsch and Beierkuhnlein 2008). There is even evidence that EWEs may favor plant introductions by reducing the resilience and/or resistance of the recipient ecosystems to invasions (Parmesan et al. 2000; Kreyling et al. 2008; Jiménez et al. 2014), although this will depend on the relative impact of the EWE on invasive and resident species (Diez et al. 2012) and the extent to which growth can be compensated at the end of the growing season (Zohner et al. 2019).

Here, we took advantage of an EWE, which was characterized by unusually low temperatures and snowfall that occurred in late February to early March 2018 over most of Northwestern Europe, to assess its impacts on invasive populations of *Gunnera tinctoria* . This invasive N‐fixing plant is associated with significant negative impacts on ecosystems in Ireland and elsewhere (Gioria and Osborne 2013) and is projected to increase in abundance and distribution with climate change (Fennell et al. 2013). However, *G. tinctoria* is thought to be restricted in its global distribution by low temperatures (Osborne and Sprent 2002) and is often protected from frosts when grown in cultivation (https://www.rhs.org.uk/plants/gunnera). Comparisons were made between uninvaded and invaded areas and with the native species *Juncus effusus* , which is often displaced by *G. tinctoria* (Mantoani et al. 2020; Mantoani and Osborne 2022).

Previously, we showed that the phenology of *G. tinctoria* enables it to initiate growth earlier than the native plant community, producing a large canopy that shades out most native species (Mantoani et al. 2020). Furthermore, we demonstrated that this same EWE acted as a removal treatment, reducing, at least temporarily (i.e., 6 months), the invader population once most leaves were killed by the extreme low temperatures and favoring the regeneration of native plants (Mantoani and Osborne 2022). To expand these results and to get a better overview of time‐dependent interactions between the EWE, *G. tinctoria* populations, and the resident community, we examined 4 years of phenological, populational, and community measurements, divided into 2 years before and 2 years after the occurrence of the EWE, known as Storm Emma. This allowed us to examine both the initial plant responses to the EWE and any subsequent recovery afterwards, including data on phenological changes, population, and community‐related responses (i.e., regeneration, plant abundance, and richness), as well as plant productivity. To the best of our knowledge, this comprises the longest empirical dataset on how an EWE impacts an invasive plant population and their time‐dependent effects.

## 2 Material and Methods

### 2.1 Experimental Design

**FIGURE 1:**
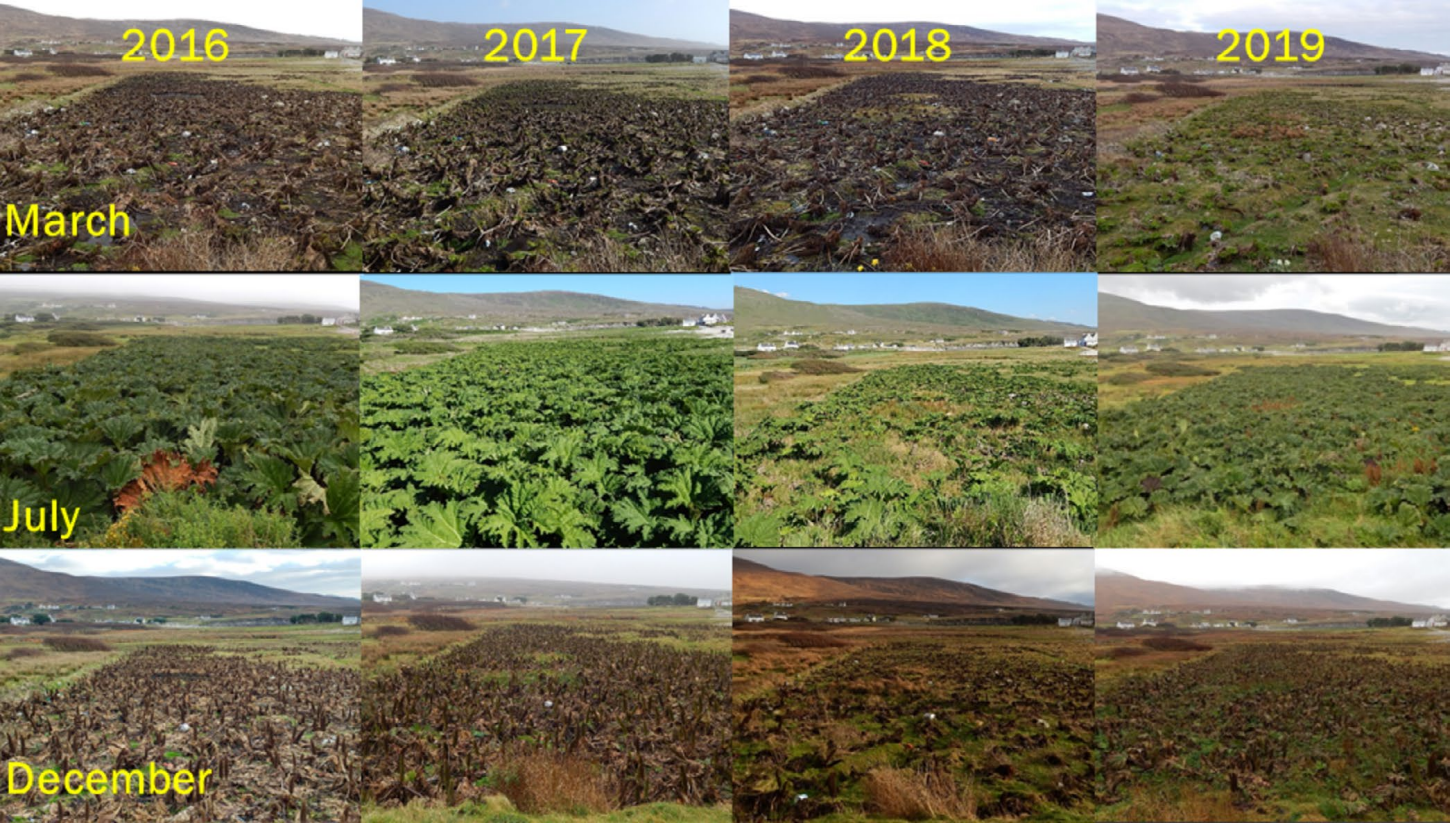
Seasonal pictures of one of the five replicate fields invaded by *G. tinctoria* that were used in the fieldwork experiment carried out on Achill Island Co. Mayo, Ireland, from 2016 to 2019. Top pictures represent spring (March; when leaves sprout from the rhizome), middle ones refer to summer (July; maximum canopy development and light interception), and bottom pictures show winter (December; shoot‐biomass senescence and decay). The years of 2016 and 2017 can be considered as “average” years, with *G. tinctoria* populations displacing the resident community due to its high biomass productivity and extensive canopy; 2018 was the year of the extreme weather event characterized by low temperatures that acted like an above‐ground removal treatment, which led to the recolonization by grassland species into invaded areas due to a decrease in light interception; in 2019, *G. tinctoria* populations showed some recovery from the negative impacts of Storm Emma, but were still smaller than previous years (2016–2017), and invaded areas had the highest number of grassland species visually seen as an increase in greening in the invaded areas.

A total of five replicate fields comprising *G. tinctoria* populations and nearby uninvaded seminatural grasslands were selected on Dooega Beach, Achill Island (53°51″ and 54°01′ N; and 9°55′ and 10°15′ W) Co. Mayo, west coast of Ireland. In each field, we established two 5×5 m plots (totalling 10 plots, *n* = 5 for each treatment) which were allocated to the following treatments: (1) uninvaded seminatural grasslands dominated by *J. effusus* ; and (2) closely associated areas invaded by *G. tinctoria* . The distance between plots was *ca*. 10 m, and each replicate field was at least 100 m apart from each other. The total area studied for this experiment was 250 m^2^ or 0.025 ha, and it encompassed 4 years of phenological, ecosystem, and environmental measurements, with empirical data collections weekly in 2016 and biweekly in 2017 until 2019. The annual rainfall is above 1100 mm, and the temperature ranges from 3°C to 6°C in winter and from 12°C to 15°C in summer (Gioria and Osborne 2013). On Achill Island, *G. tinctoria* invades alluvial or colluvial soils, derived from volcanic material or thin gley soils of marine origin, with a pH that ranges from 4.6 to 6.2 (Gioria and Osborne 2013). Figure 1 and Figures S1 and S2 provide pictures of the fieldwork experiment, showing the plots, measurements of individuals of *G. tinctoria* and *J. effusus* , the environmental variables collected, and the impact of the EWE on seedling development.

### 2.2 Phenological Assessments, Plant Parameters, and Environmental Variables

As per Mantoani et al. (2020, 2022) and Mantoani and Osborne (2021, 2022), we assessed the phenology of *G. tinctoria* and *J. effusus* with a description of their major phenophases (e.g., leaf‐out, development of inflorescences). We also collected data on a number of plant parameters, including total leaf number and area (for the leaf area equations please refer to Mantoani et al. 2020), petiole length (measured from the insertion point on the rhizome to the basis of each leaf), and thickness (measured with a caliper scale at the median part of the petiole), inflorescence number and size (measured from the insertion point on the rhizome until the tip of the organ), as well as plant height (estimated with the aid of a metallic tape measuring the distance between the tip of the highest leaf to the ground). In conjunction with the phenological assessments, soil moisture and temperature data were collected using a handheld WET‐2 WET sensor (Delta‐T Devices Ltd., 2007, Cambridge, England) at three random points within the plots, with values being averaged for each replicate. Measurements of light interception, the proportion of photosynthetically active radiation (400–700 nm) underneath the canopy of *G. tinctoria* relative to that of a fully exposed area, was determined using a SpectroSense 2 sensor (Skye Instruments Ltd., 2007, Llandrindod Wells, Powys, UK) at 16 random points that were subsequently averaged to provide one value for each replicate.

### 2.3 Biomass Productivity and Number of Species Regenerating in the Plots

Biomass productivity was determined by harvesting plants at the end of the growing season in October, followed by drying it in a forced draft oven at 70°C until constant weight. For invaded plots, we harvested one individual adjacent to the plots, multiplied its weight by the number of mature *G. tinctoria* in the plots (25–30) and divided it by the plot area (25 m^2^; an average of 1.1 *G. tinctoria* rhizomes per plot) to obtain the biomass per m^2^. To estimate the biomass of uninvaded grasslands, we harvested 1 m^2^ in areas adjacent to uninvaded plots and separated the biomass into *J. effusus* and other grassland species. The total above‐ground biomass for both invaded and uninvaded areas was also separated into shoot biomass (considering only *J. effusus* for the uninvaded grasslands and leaves, petioles and inflorescences for *G. tinctoria* ). To compare the number of species regenerating in uninvaded grasslands and invasive stands, following Mantoani and Osborne (2022), an inventory of all plants accounting specifically for the number of species (i.e., richness, presence or absence only) was also performed at the end of each growing season from 2016 to 2019. The species identified are listed in the Table S1.

### 2.4 Estimations of Seed Production Loss for 2018

To estimate the effect of the EWE on seed production, the following calculations were done. As *G. tinctoria* has been estimated to produce more than 200,000 seeds per plant (Gioria and Osborne 2013) and given that there are *ca*. 11,000 plants per hectare (Mantoani et al. 2020; Mantoani and Osborne 2022; 1.1 plant per m^2^), invasive populations could potentially produce 2.2 billion seeds per hectare. Since only 10% of the plants produced viable inflorescences after the EWE in 2018 (see below), that would represent a drop of 1.98 billion seeds per hectare due to Storm Emma's occurrence.

### 2.5 *Gunnera tinctoria* Seedlings

In order to assess any differences between mature plants and seedlings, as documented before (Mantoani and Osborne 2021, 2022), we cleared 5 × 5 m patches in the middle of invasive stands in October 2016. All mature plants of *G. tinctoria* were removed to allow plant regeneration. As seed germination does not occur during winter (Gioria and Osborne 2013), any seedlings that appeared would be less than 1 year of age during the initial measurements, reflecting the early stages of establishment. After seedling emergence, in October 2017, we marked seven individuals (*n* = 7, for seedlings) for phenological measurements, which were made on a biweekly basis, as we did for mature plants. Thus, although we cannot be precise about the age of the different seedlings, they were all less than 1 year of age at the start of the phenological assessments in October 2017, and up to 3 years of age by the time of the last measurements in December 2019.

### 2.6 Storm Emma Characterization

The low temperature EWE had its largest impact in Ireland from late February to early March 2018, bringing widespread snow and record low temperatures. A region of high pressure over Scandinavia caused an exceptionally cold air mass to move over Ireland from Siberia. This blocking high caused a low‐pressure storm, called Storm Emma, to bring heavy rain to Portugal and Spain before moving northwards to Ireland. As the moisture‐rich air from Storm Emma met the cold air mass over Ireland, heavy snowfall occurred across much of the country. Record low temperatures and ice days (where the maximum temperature does not rise above freezing) were recorded on the 1st of March in the region of this study (based on Knock Airport data Co. Mayo). This is the first‐time ice days were recorded in March for any station in Ireland since digitized records began in 1942 (Met Éireann 2019).

### 2.7 Assessments of Past and Future Occurrence of EWEs

To examine the occurrence of past EWEs of a similar or greater magnitude to Storm Emma, we used data from the weather station at Belmullet, Co. Mayo, as this is the closest synoptic station with a long record (1957 to 2023) and a complete dataset of daily maximum and daily minimum air temperatures. Historical daily temperature data were downloaded from Met Éireann, Ireland's National Meteorological Service (www.met.ie). To examine the temperature anomalies associated with this event, we first generated climate values based on a 31‐day rolling mean of the daily data for the 30‐year period from 1971 to 2000 and then calculated the average for each day of the year. This 30‐year period was chosen so that it overlapped the EURO‐CORDEX historical data, which is only available up to 2005. The climate values for each day of the year were then subtracted from the daily temperature values for the full time series (1957–2023) to give daily temperature anomaly values.

For future projections, we downloaded data from the EURO‐CORDEX Climate data store. The model data was used from the ichec_ec_earth Global Climate Model, downscaled to 0.11° by the mohc_hadrem3_ga7_05 Regional Climate Model. Data from the closest land model grid point to the Belmullet station location were used. The same method as described above was used to calculate the climate values for the Climate model historical data from 1971 to 2000. Temperature anomalies were then calculated for the low and high IPCC scenarios, RCP2.6 and RCP8.5, for the period 2070–2100.

### 2.8 Statistical Analysis

As per Mantoani et al. (2020, 2022) and Mantoani and Osborne (2021, 2022), general linear mixed‐model effects analyses with restricted maximum likelihood were used to verify differences among treatments and their interaction with sampling times, considering treatments as fixed factors and fields as random factors. Bonferroni's *post hoc* correction pair‐wise comparisons were performed to evaluate differences in light interception, total biomass productivity and the number of species between uninvaded seminatural grasslands and invasive stands, as well as differences in total leaf area between *G. tinctoria* and *J. effusus* . Analysis of Variance (ANOVA) followed by Bonferroni's *post hoc* correction was used to check differences between the lengths of the growing season among the 4 years (2016–2019). Data on light interception was arcsine square root transformed prior to analysis, and visual analysis of the residuals was also carried out to ensure normality. All analyses were performed with a significance level of *p =* 0.05, using SPSS Statistics v. 24.

## 3 Results

### 3.1 Growth, Phenology, and Biomass of Invasive Stands

**FIGURE 2:**
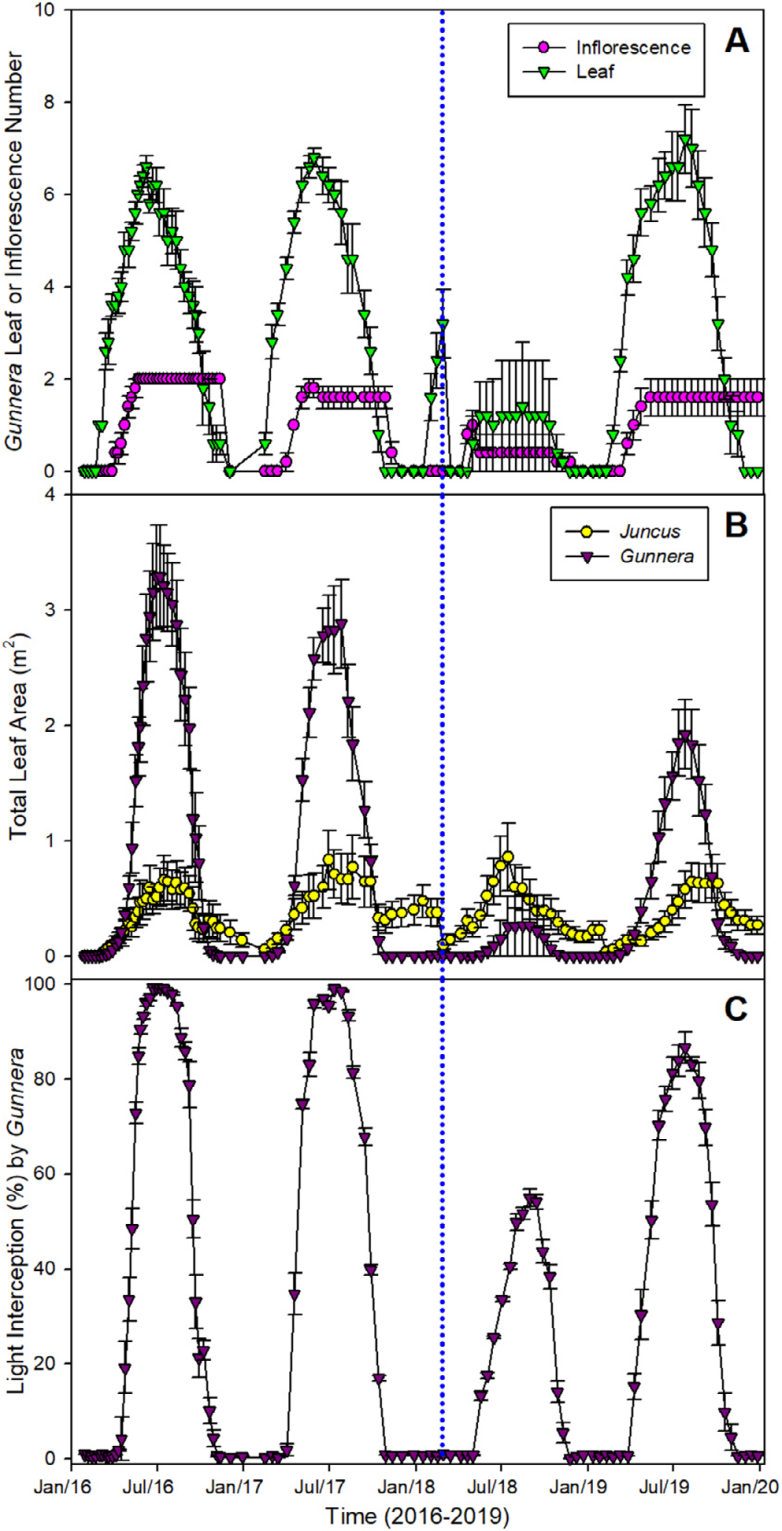
Seasonal variation in different growth‐related parameters for *G. tinctoria* plants growing on Achill Island Co. Mayo, Ireland from 2016 to 2019 (*n* = 5; mean ± 1SE). (A) Total leaf and inflorescence number for *G. tinctoria* only; (B) total leaf area (m^2^) for *J. effusus* (*Juncus*) and *G. tinctoria* (*Gunnera*); and (C) light interception by *G. tinctoria* (%). The vertical dotted line represents the occurrence of the extreme weather event called Storm Emma, in late February early March 2018.

Although varying amongst years (Figure 2A), the total number of leaves produced by *G. tinctoria* in 2018 (6.80 ± 1.28), the year of the low‐temperature EWE, was not significantly different from 2019 (10.20 ± 0.86), the year after the event, 2017 (8.20 ± 0.20) or 2016 (8.80 ± 0.49; *F*_(3,16)_ = 2.84; *p =* 0.071). After the EWE, the maximum total leaf area of *G. tinctoria* was 10‐ (95% CI = −3.09, −2.13) to 12‐fold (95% CI = −3.50, −2.55; *F*_(110,888)_ = 15.11; *p <* 0.001; Figure 2B) smaller when compared to 2017 or 2016. In 2018, the maximum total leaf area of *G. tinctoria* (0.266 ± 0.266 m^2^; 95% CI = −1.12, −0.09) was 3‐fold smaller than that of *J. effusus* (0.859 ± 0.294 m^2^), when typically, it is 3‐to‐5‐fold higher (Figure 2B). Consequently, the point at which 50% of the light was intercepted by the *G. tinctoria* canopy was delayed for 90 days, and the maximum light interception was 55%, compared with nearly 100% in previous years (*F*_(93,752)_ = 317.50; *p <* 0.001; Figure 2C). Nevertheless, in the following year after the EWE, *G. tinctoria* had a light interception of > 85% by July, despite a smaller leaf area (*ca*. 2 m^2^) when compared to the years before Storm Emma. The maximum heights of *G. tinctoria* plants during the summer period prior to Storm Emma were greater than 2 m (Figure S3A). In 2019, after the EWE occurrence, they were reduced to approximately 50 cm, which was *ca*. 65% of that in previous years, subsequently increasing to *ca*. 130 cm in the summer of 2019. Total petiole length and thickness both paralleled these changes (Figure S3B,C), reaching *ca*. 70% and *ca*. 68% of their average values, respectively, in 2019 in comparison with the years before the EWE.

**FIGURE 3:**
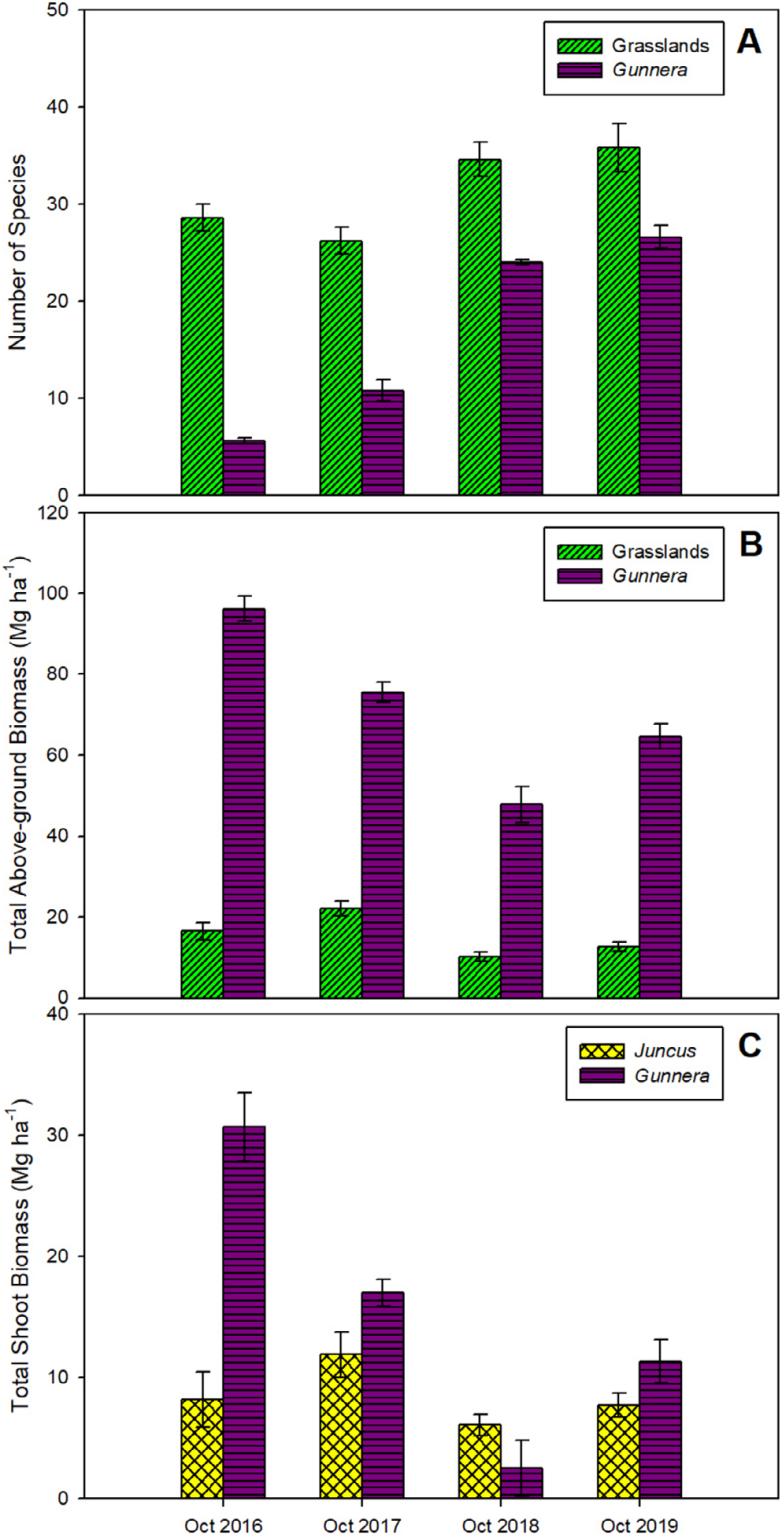
Variation in the number of species (A) and total above‐ground biomass productivity (B), for uninvaded seminatural grasslands (Grasslands) and areas invaded by *G. tinctoria* (*Gunnera*), and the total shoot biomass (C) for *J. effusus* (*Juncus*) and *G. tinctoria* (leaves, petioles and inflorescences), in October, at the end of the growing season. Measurements were made from 2016 to 2019, on plants growing on Achill Island Co. Mayo, Ireland (*n* = 5; mean ± 1SE).

Concomitant with the reductions in leaf area and light interception, there was a 4‐fold increase in the number of species that emerged in invasive stands (*F*_(3,32)_ = 9.98; *p <* 0.001; Figure 3A) in 2018 (19.4; 95% CI = 15.34, 23.46) and 2019 (21.0; 95% CI = 16.94, 25.06), compared to those found in the years before Storm Emma. This was also accompanied by a reduction in the total above‐ground biomass (*F*_(3,32)_ = 9.04; *p <* 0.001; Figure 3B), with 35%–50% less biomass in invaded areas in 2018, in comparison with 2016 (95% CI = −60.23, −36.54) and 2017 (95% CI = −39.62, −15.91). This was largely due to a reduction in *G. tinctoria* shoot biomass in 2018 (*F*_(3,32)_ = 17.17; *p <* 0.001; Figure 3C), with an 85% (95% CI = −19.91, −9.03) and 92% (95% CI = −33.54, −22.72) lower shoot biomass in comparison with 2017 and 2016, respectively.

In 2018, most of the inflorescences (83%) were killed or severely damaged after the EWE (Figure 2A). The average length of *G. tinctoria* inflorescences went from *ca*. 75 cm in 2016–2017 to 10 cm in 2018. Based on the numbers of inflorescences lost and the average number of seeds per inflorescence, this could represent a reduction in nearly 2 billion seeds per hectare (see Methods section). After the EWE, there was a significant recovery of plant biomass and inflorescence size to values that were 67%–86% of previous years. In comparison, the total biomass of the uninvaded grasslands (Figure 3B), or the biomass of *J. effusus* (Figure 3C), was unaffected by the EWE.

### 3.2 Growth and Phenology of Seedlings

**FIGURE 4:**
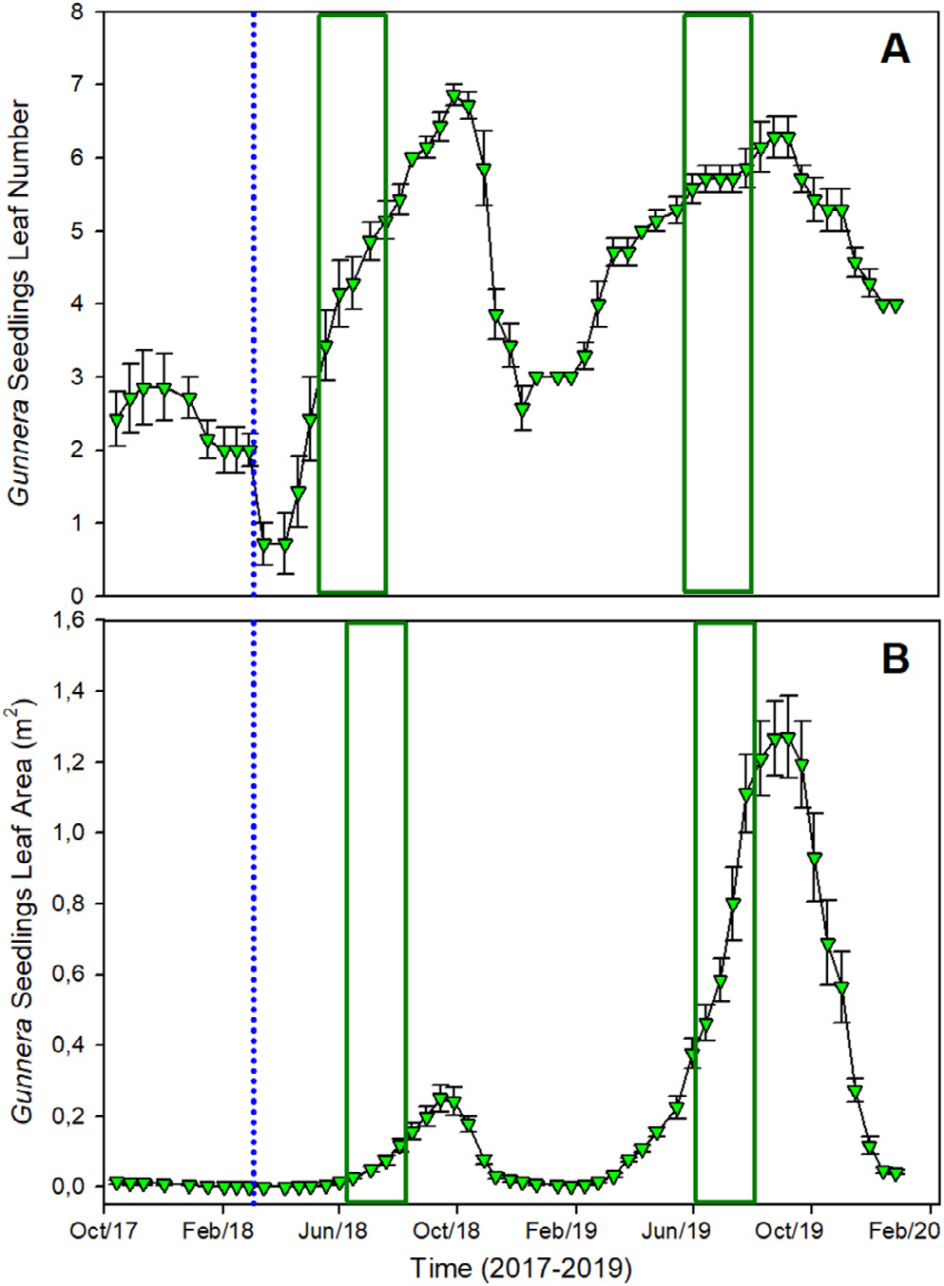
Seasonal variation in (A) the total leaf number of seedlings and (B) the total leaf area (m^2^), of seedlings of populations growing on Achill Island Co. Mayo, Ireland from 2017 to 2019 (*n* = 7; mean ± 1SE). The vertical dotted line represents the occurrence of the extreme weather event called Storm Emma, in late February early March 2018. The green boxes indicate the period where there was the highest number of leaves or the largest leaf area for mature *Gunnera* plants, in each year.

While the mortality of mature *G. tinctoria* plants was high (up to 40%—see Mantoani and Osborne 2022), all the marked seedlings survived the EWE. Not only did the *G. tinctoria* seedlings retain their leaves during the winter of 2018 (Figure 4A), but leaf number also increased by 7‐fold, from approximately one leaf per seedling in winter to seven leaves in the following summer, with leaf area increasing by more than 1 m^2^ within 1 year (Figure 4B). The growth of seedlings also peaked later in the year, with the total leaf area and total leaf numbers reaching their highest in September, whereas this occurred in the middle of July for mature *G. tinctoria* plants.

### 3.3 Environmental Conditions and Characterization of Past and Future EWEs

**FIGURE 5:**
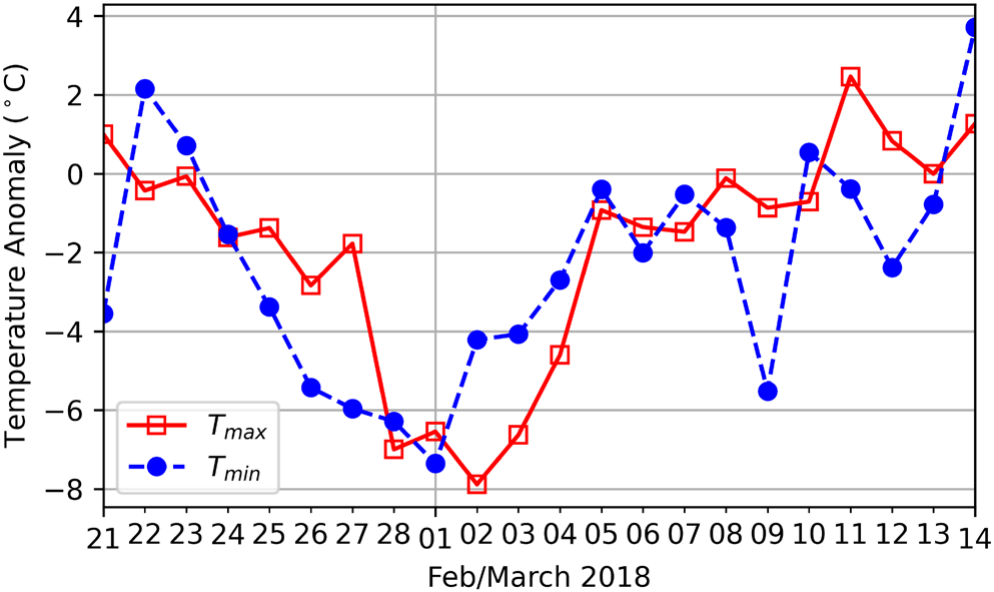
Daily maximum (*T*_max_) and daily minimum (*T*_min_) temperature anomaly values at Belmullet during the Storm Emma EWE.

Soil temperature and moisture varied significantly throughout the years and seasons (Figure S4). For temperature, the largest range (0°C–30°C) was found during the year of the EWE, and the lowest soil moisture contents (*ca*. 27%) occurred during the summer of 2019. Daily maximum and minimum temperature anomalies (*T*_max_, *T*_min_) for the period associated with Storm Emma (24 February to 5 March 2018) are shown in Figure 5. Based on this data, the plant populations were exposed to negative temperature anomalies for over 14 days, with the largest anomaly for *T*_min_ on the 1st of March and the largest T_max_ on the 2nd of March. To use this information for estimating the occurrence of past and future EWEs, we characterized Storm Emma as an event with both daily *T*_max_ and daily *T*_min_ anomalies < −4°C for a 4‐day period or more, occurring in the months of January, February, or March. In total, during the period 1957–2023, 13 such EWEs were identified (or an average of 1.9 events per decade).

To determine the likely occurrence of similar events in the future, we first used historical climate model data for the 1971–2000 period using the same < −4°C criterion for temperature anomalies. A total of five EWEs were identified in this climate model data, giving around 1.7 events per decade. Then, we used the climate model projection data to calculate the occurrence of EWEs for the IPCC low (RCP 2.6) and high (RCP 8.5) future emission scenarios. For the RCP 2.6 scenario, only one EWE occurred during the 30‐year period 2071–2100, whereas none occurred for the RCP 8.5 scenario.

## 4 Discussion

The production of an extensive canopy and reductions in light interception are among the main negative effects on resident species caused by many large plant invaders such as *G. tinctoria* (Gioria and Osborne 2013; Mantoani et al. 2020) and Japanese knotweed (Groeneveld et al. 2014). Reductions in light level, due to the formation of an extensive canopy, are presumably the major reason why few resident species emerge from stands of *G. tinctoria* and many other large invasive plant species. Consequently, Storm Emma, by reducing canopy development and light interception, led to a 5‐fold higher number of resident plant species in invaded areas. Interestingly, the number of resident species remained high even after the almost complete recovery of the plant canopy with light interception values of > 80%. There could be several reasons for this, including: (1) complete canopy closure is required to eliminate seedling emergence from the soil seed bank; (2) shading has a greater impact on seedling germination and establishment; and (3) complete canopy cover may be required to physically prevent the input of seed from surrounding areas.

The results from this and other studies indicate that at least some invasive species are, albeit temporarily, more susceptible to low temperature extremes than other species (Bykova and Sage 2012; Dainese et al. 2017; Song 2017), despite reports to the contrary (Yue et al. 2021; Maloney et al. 2022) including evidence suggesting an increase in chilling tolerance (Fenollosa and Munné‐Bosch 2019). Based on the results of the current study, a reduction in leaf expansion because of Storm Emma is the major reason for the reduced canopy development, although the initiation of fewer leaves is also a contributory factor. Whilst the numbers of inflorescences were also reduced, it was of a smaller extent, indicating that the longer‐term impacts of Storm Emma would be on vegetative growth rather than on reproductive capacity. Even though we estimated a dramatic reduction in seed production, this was again only temporary, with almost complete recovery of the number and size of inflorescences 1 year after the EWE, and this would have little impact on plant establishment from seed given *G. tinctoria*'s large and viable seed bank (Gioria and Osborne 2013).

It has previously been suggested that environmental constraints on leaf expansion that limit the performance *of G. tinctoria* may be related to its poor control of water balance, which could be exacerbated by low temperatures, as large amounts of water are required for the growth of leaves (Osborne et al. 1991; Osborne and Sprent 2002), some of which may be > 2 m^2^ in size. A consequence of the results of the current study is that plant communities that are impacted by the presence of some introduced species may benefit from the effects of climate change‐related low temperature EWEs. However, *G. tinctoria* plants showed significant recovery in terms of canopy development and the number and size of the inflorescences within 1 year after the EWE, indicating that any effects are likely to be temporary. Clearly, however, the recovery of the invasive population could depend on the magnitude, timing, and frequency of the EWE (Diez et al. 2012), as well as on any growth compensation that might occur later in the growing season (Zohner et al. 2019).

Although the effects of an EWE on the productivity of invaded communities are equivocal (Jentsch and Beierkuhnlein 2008; Diez et al. 2012), our results reinforce the idea that some invasive species are negatively impacted by an EWE while native species are much more resilient (Reyer et al. 2013). Whilst *G. tinctoria* was severely impacted by the EWE, *J. effusus* and the resident community in general were unaffected. The number of species increased, and the biomass remained comparable to other years in the uninvaded grasslands. Whilst we cannot separate the effects of early growth from a greater resilience to episodic low temperature events, by starting growth later, native species may avoid the impacts of an EWE that occurs early in the year (Zohner and Renner 2017), which could partially explain why *J. effusus* was not impacted by Storm Emma. Thus, growing earlier may not always represent a benefit given the risks associated with exposure to spring frosts (Laube et al. 2015; Vitasse et al. 2018), highlighting that climate change‐related extension in the growing period will not always benefit invasive plants as often suggested (Polgar and Primack 2011; Wolkovich and Cleland 2011; Fridley 2012; Wolkovich et al. 2013; Groeneveld et al. 2014; Polgar et al. 2014; Gallinat et al. 2015; Ratcliffe et al. 2024).

The evidence from this study indicates that these long‐lived populations of *G. tinctoria* have been exposed to low temperature EWEs of a similar or greater magnitude in the past with little evidence that this has had any significant longer‐term impact (Fennell et al. 2013). Previous suggestions that *G. tinctoria* may be limited by low temperatures based on its global distribution (Osborne 1989; Osborne et al. 1991; Osborne and Sprent 2002) or information from the horticultural trade (https://www.rhs.org.uk/plants/gunnera) that suggests this species needs to be protected from low temperature extremes are probably biased towards the immediate and potentially dramatic effects of reduced temperatures on above‐ground biomass and do not account for an ability to rapidly recover afterwards. The occurrence of *G. tinctoria* as a colonizer of glacial moraines in its native range in the Andes of Chile (Piper, pers. comm.) also indicates that the longer‐term persistence of this species may not be as sensitive to low temperatures as once thought.

Based on the results for the native species, *J. effusus*, which was largely unaffected by the EWE, short‐term studies that fail to account for recovery after an EWE or other environmental perturbation may lead to erroneous conclusions about how this influences the dominance or persistence of resident or introduced species. Based on our analysis, the likelihood of similar EWEs occurring in the future is small, suggesting that this would remove even any temporary restrictions on the growth and reproduction of established populations of *G. tinctoria* . It remains to be seen, however, whether the limited removal of above‐ground plant parts, as would occur during a low‐temperature EWE, would increase rather than decrease plant growth and reproduction (Liu et al. 2015), emphasizing the need for longer‐term studies on invasive populations.

There were clear differences between the effects of Storm Emma on mature *G. tinctoria* plants and their seedlings. Surprisingly, seedlings were more resilient to the EWE than might have been expected (Gioria and Osborne 2013). We attribute this in part to differences in the growth and development of seedlings compared to mature plants. Seedlings of *G. tinctoria* initiate growth later in the year in comparison to mature plants (Figure 4). They can also retain, as well as produce new leaves over winter, indicating that their growth and development may not be as constrained by low temperatures as mature plants. This may be due, in part, to the lower water requirements associated with the expansion of these smaller leaves compared to mature plants, given their high‐water demand and the possibility that low temperatures might restrict the uptake and/or transport of water in this species (Osborne et al. 1991; Osborne and Sprent 2002).

The later growth and development of seedlings may be important for early establishment by exploiting a period when the resident species are largely inactive and when light interception by the mature plants is low. Importantly, this could be an advantage for introduced species with a poor competitive ability and facilitate the early growth of established plants the following year prior to that of resident species (Gioria et al. 2018). This could also be important in largely avoiding the major effects of an EWE or early spring frosts that could be detrimental to their growth. While late growth is a feature of many invasive species (Fridley 2012; Gallinat et al. 2015) and the differences in growth and development may also explain the success of some invaders (Ni et al. 2018), our results show that phenological differences may also contribute to the avoidance of growth limiting conditions during the early establishment of seedlings.

Past increases in the length and an earlier start to the growing season in Ireland (Nolan and Flanagan 2014) may have benefited an early growing species like *G. tinctoria* , as our results also show that this EWE only has a temporary impact on plant growth and development. In addition, our findings of the near absence of future low temperature EWEs are in line with the findings of the IPCC AR6 WG1 report (Arias et al. 2021; Tables S2 and S5), which indicate that cold extremes are virtually certain to decrease in future projections for warming levels from 1.5°C to 4°C. The net effect of a low temperature EWE on *G. tinctoria* populations will, however, depend on the timing, magnitude, and frequency of EWEs and how quickly the introduced species will recover. Given that mature *G. tinctoria* plants recovered significantly after only 1 year and that its seedlings were largely unaffected by the EWE, current and future invasions by this alien plant are unlikely to be significantly impacted by low temperature EWEs. On the other hand, as indicated previously, an EWE‐related impairment of growth does provide an opportunity for the more effective implementation of control measures (Bradley et al. 2010; Diez et al. 2012) particularly for large and difficult‐to‐eradicate invasive species, such as *G. tinctoria* , and this would require the close monitoring of invaded areas.

## Author Contributions

**Maurício Cruz Mantoani:** conceptualization, data curation, formal analysis, funding acquisition, investigation, project administration, resources, software, validation, visualization, writing – original draft, writing – review and editing. **Conor Sweeney:** data curation, formal analysis, methodology, software, validation, visualization, writing – original draft, writing – review and editing. **Bruce A. Osborne:** conceptualization, data curation, formal analysis, funding acquisition, investigation, methodology, project administration, resources, software, supervision, validation, visualization, writing – original draft, writing – review and editing.

## Conflicts of Interest

The authors declare no conflicts of interest.

## Supporting information


Data S1.

## Data Availability

The data that support the findings of this study are openly available in Zenodo at https://doi.org/10.5281/zenodo.14899894. Meteorological observation data were obtained from Met Éireann, Ireland's National Meteorological Service at https://www.met.ie/climate/available‐data/historical‐data. EURO‐CORDEX climate model data were obtained from the Copernicus Climate Change Service (C3S) Climate Data Store (CDS) at https://doi.org/10.24381/cds.bc91edc3.

## References

[gcb70113-bib-0001] Allan, R. P. , and B. J. Soden . 2008. “Atmospheric Warming and the Amplification of Precipitation Extremes.” Science 321: 1481–1484. 18687921 10.1126/science.1160787

[gcb70113-bib-0002] Arias, P. A. , N. Bellouin , E. Coppola , et al. 2021. “Technical Summary.” In Intergovernmental Panel on Climate Change Climate Change 2021: The Physical Science Basis. Contribution of Working Group I to the Sixth Assessment Report of the Intergovernmental Panel on Climate Change, edited by V. Masson‐Delmotte , P. Zhai , A. Pirani , et al., 33–144. Cambridge University Press.

[gcb70113-bib-0003] Bradley, B. A. , D. M. Blumenthal , D. S. Wilcove , and L. H. Ziska . 2010. “Predicting Plant Invasions in an Era of Global Change.” Trends in Ecology & Evolution 25: 310–318. 20097441 10.1016/j.tree.2009.12.003

[gcb70113-bib-0004] Bykova, O. , and R. F. Sage . 2012. “Winter Cold Tolerance and the Geographic Range Separation of Bromus Tectorum and *Bromus rubens*, Two Severe Invasive Species in North America.” Global Change Biology 18: 3654–3663.

[gcb70113-bib-0005] Cook, B. I. , E. M. Wolkovich , and C. Parmesan . 2012. “Divergent Responses to Spring and Winter Warming Drive Community Level Flowering Trends.” Proceedings of the National Academy of Sciences 109: 9000–9005. 10.1073/pnas.1118364109 PMC3384199 22615406

[gcb70113-bib-0006] Dainese, M. , S. Aikio , P. E. Hulme , A. Bertolli , F. Prosser , and L. Marini . 2017. “Human Disturbance and Upward Expansion of Plants in a Warming Climate.” Nature Climate Change 7: 577–580.

[gcb70113-bib-0007] Diez, J. M. , C. M. D'Antonio , J. S. Dukes , et al. 2012. “Will Extreme Climatic Events Facilitate Biological Invasions?” Frontiers in Ecology and the Environment 10: 249–257.

[gcb70113-bib-0008] Easterling, D. R. , G. A. Meehl , C. Parmesan , S. A. Changnon , T. R. Karl , and L. O. Mearns . 2000. “Climate Extremes: Observations, Modeling, and Impacts.” Science 289: 2068–2074. 11000103 10.1126/science.289.5487.2068

[gcb70113-bib-0009] Éireann, M. 2019. “Technical Report. Storm Emma, an Analysis of Storm Emma and the Cold Spell Which Struck Ireland Between the 28th of February and the 4th of March 2018.” https://www.met.ie/cms/assets/uploads/2019/02/EmmaReport2019.pdf.

[gcb70113-bib-0010] Fennell, M. , J. E. Murphy , T. Gallagher , and B. Osborne . 2013. “Simulating the Effects of Climate Change on the Distribution of an Invasive Plant, Using a High Resolution, Local Scale, Mechanistic Approach: Challenges and Insights.” Global Change Biology 19: 1262–1274. 23504901 10.1111/gcb.12102

[gcb70113-bib-0011] Fenollosa, E. , and S. Munné‐Bosch . 2019. “Increased Chilling Tolerance of the Invasive Species *Carpobrotus edulis* May Explain Its Expansion Across New Territories.” Conservation Physiology 7: coz075. 10.1093/conphys/coz075. 31737274 PMC6846103

[gcb70113-bib-0012] Fridley, J. D. 2012. “Extended Leaf Phenology and the Autumn Niche in Deciduous Forest Invasions.” Nature 485: 359–362. 22535249 10.1038/nature11056

[gcb70113-bib-0013] Gallinat, A. S. , R. B. Primack , and D. L. Wagner . 2015. “Autumn, the Neglected Season in Climate Change Research.” Trends in Ecology & Evolution 30: 169–176. 25662784 10.1016/j.tree.2015.01.004

[gcb70113-bib-0014] Gioria, M. , and B. Osborne . 2013. “Biological Flora of the British Isles: *Gunnera tinctoria* .” Journal of Ecology 101: 243–264.

[gcb70113-bib-0015] Gioria, M. , P. Pyšek , and B. A. Osborne . 2018. “Timing Is Everything: Does Early and Late Germination Favor Invasions by Herbaceous Alien Plants?” Journal of Plant Ecology 11: 4–16.

[gcb70113-bib-0016] Groeneveld, E. , F. Belzile , and A. C. Lavoie . 2014. “Sexual Reproduction of Japanese Knotweed (*Fallopia japonica* s.l.) at Its Northern Distribution Limit: New Evidence of the Effect of Climate Warming on an Invasive Species.” American Journal of Botany 101: 459–466. 24567127 10.3732/ajb.1300386

[gcb70113-bib-0017] Jentsch, A. , and C. C. R. Beierkuhnlein . 2008. “Research Frontiers in Climate Change: Effects of Extreme Meteorological Events on Ecosystems.” Geoscience 340: 621–628.

[gcb70113-bib-0018] Jentsch, A. , J. Kreyling , J. Boettcher‐Treschkow , and C. Beierkuhnlein . 2009. “Beyond Gradual Warming: Extreme Weather Events Alter Flower Phenology of European Grassland and Heath Species.” Global Change Biology 15: 837–849.

[gcb70113-bib-0019] Jiménez, M. A. , F. M. Jaksic , J. J. Armesto , et al. 2014. “Extreme Climatic Events Change the Dynamics and Invasibility of Semi‐Arid Annual Plant Communities.” Ecology Letters 14: 1227–1235. 10.1111/j.1461-0248.2011.01693.x 21988736

[gcb70113-bib-0020] Kreyling, J. , C. Beierkuhnlein , L. Ellis , and A. Jentsch . 2008. “Invasibility of Grassland and Heath Communities Exposed to Extreme Weather Events: Additive Effects of Diversity Resistance and Fluctuating Physical Environment.” Oikos 117: 1542–1554.

[gcb70113-bib-0021] Laube, J. , T. H. Sparks , C. Bässler , and A. Menzel . 2015. “Small Differences in Seasonal and Thermal Niches Influence Elevational Limits of Native and Invasive Balsams.” Biological Conservation 191: 682–691.

[gcb70113-bib-0022] Liu, T. , L. Gu , S. Dong , J. Zhang , P. Liu , and B. Zhao . 2015. “Optimum Leaf Removal Increases Canopy Apparent Photosynthesis, ^13^C‐Photosynthate Distribution and Grain Yield of Maize Crops Grown at High Density.” Field Crops Research 170: 32–39.

[gcb70113-bib-0023] Maloney, M. E. , A. Hay , E. B. Borth , and R. W. McEwan . 2022. “Leaf Phenology and Freeze Tolerance of the Invasive Tree *Pyrus calleryana* (Roseaceae) and Potential Native Competitors.” Journal of the Torrey Botanical Society 149: 273–279.

[gcb70113-bib-0024] Mantoani, M. C. , F. T. Alhakami , H. Fearon , M. Gioria , O. Schmidt , and B. A. Osborne . 2022. “ *Gunnera tinctoria* Invasions Increase, Not Decrease, Earthworm Abundance and Diversity.” Biological Invasions 24: 3721–3734.

[gcb70113-bib-0025] Mantoani, M. C. , A. B. González , L. G. Sancho , and B. A. Osborne . 2020. “Growth, Phenology and N‐Utilization by Invasive Populations of *Gunnera tinctoria* .” Journal of Plant Ecology 13: 589–600.

[gcb70113-bib-0026] Mantoani, M. C. , and B. A. Osborne . 2021. “Alien Plant Introductions and Greenhouse Gas Emissions: Insights From *Gunnera tinctoria* Invasions.” Science of the Total Environment 775: 145861. 33621871 10.1016/j.scitotenv.2021.145861

[gcb70113-bib-0027] Mantoani, M. C. , and B. A. Osborne . 2022. “Post‐Invasion Recovery of Plant Communities Colonised by *Gunnera tinctoria* After Mechanical Removal or Herbicide Application and Its Interaction With an Extreme Weather Event.” Plants 11: 1224. 35567226 10.3390/plants11091224 PMC9104690

[gcb70113-bib-0028] Ni, M. , Y. Liu , C. Chu , H. Xu , and S. Fang . 2018. “Fast Seedling Root Growth Leads to Competitive Superiority of Invasive Plants.” Biological Invasions 20: 1821–1832.

[gcb70113-bib-0029] Nolan, P. , and J. Flanagan . 2014. “High‐Resolution Climate Projections for Ireland—A Multi‐Model Ensemble Approach (2014‐CCRP‐MS.23).” EPA Research Report, 37.

[gcb70113-bib-0030] Osborne, B. 1989. “Effect of Temperature on Photosynthetic Oxygen‐Exchange and Slow Fluorescence Characteristics of *Gunnera tinctoria* (Molina) Mirbel.” Photosynthetica 23: 77–88.

[gcb70113-bib-0031] Osborne, B. A. , F. Doris , A. Cullen , R. McDonald , G. Campbell , and M. Steer . 1991. “ *Gunnera tinctoria*: An Unusual Nitrogen‐Fixing Invader.” Bioscience 41: 224–234.

[gcb70113-bib-0032] Osborne, B. A. , and J. I. Sprent . 2002. “Ecology of the *Nostoc‐Gunnera* Symbiosis.” In Cyanobacteria in Symbiosis, edited by A. Rai , B. Bergman , and U. Rasmussen , 233–251. Academic Press.

[gcb70113-bib-0033] Parmesan, C. , T. L. Root , and M. R. Willig . 2000. “Impacts of Extreme Weather and Climate on Terrestrial Biota.” Bulletin of the American Meteorological Society 87: 443–450.

[gcb70113-bib-0034] Polgar, C. A. , A. Gallinat , and R. B. Primack . 2014. “Drivers of Leaf‐Out Phenology and Their Implications for Species Invasions: Insights From Thoreau's Concord.” New Phytologist 202: 106–115. 24372373 10.1111/nph.12647

[gcb70113-bib-0035] Polgar, C. A. , and R. B. Primack . 2011. “Leaf‐Out Phenology of Temperate Woody Plants: From Trees to Ecosystems.” New Phytologist 191: 926–941. 21762163 10.1111/j.1469-8137.2011.03803.x

[gcb70113-bib-0036] Ratcliffe, H. , A. Kendig , S. Vacek , D. Carlson , M. Ahlering , and L. E. Dee . 2024. “Extreme Precipitation Promotes Invasion in Managed Grasslands.” Ecology 105: e4190. 10.1002/ecy.4190. 37877294

[gcb70113-bib-0037] Reyer, C. P. O. , S. Leuzinger , A. Rammig , et al. 2013. “A Plant's Perspective of Extremes: Terrestrial Plant Responses to Changing Climatic Variability.” Global Change Biology 19: 75–89. 23504722 10.1111/gcb.12023 PMC3857548

[gcb70113-bib-0038] Song, U. 2017. “Temperature‐Dependent Performance of Competitive Native and Alien Invasive Plant Species.” Acta Oecologica 84: 8–14.

[gcb70113-bib-0039] Vitasse, Y. , L. Schneider , C. Rixen , D. Christen , and M. Rebetez . 2018. “Increase in the Risk of Exposure of Forest and Fruit Trees to Spring Frosts at Higher Elevations in Switzerland Over the Last Four Decades.” Agricultural and Forest Meteorology 248: 60–69.

[gcb70113-bib-0040] Wolkovich, E. M. , and E. E. Cleland . 2011. “The Phenology of Plant Invasions: A Community Ecology Perspective.” Frontiers in Ecology and the Environment 9: 287–294.

[gcb70113-bib-0041] Wolkovich, E. M. , T. J. Davies , H. Schaefer , et al. 2013. “Temperature‐Dependent Shifts in Phenology Contribute to the Success of Exotic Species With Climate Change.” American Journal of Botany 100: 1407–1421. 23797366 10.3732/ajb.1200478

[gcb70113-bib-0042] Yue, M. , H. Shen , W. Ye , W. Li , and J. Chen . 2021. “Winter Low Temperature Disturbance in the Southern Subtropics of China Promotes the Competitiveness of an Invasive Plant.” Biological Invasions 23: 2913–2925.

[gcb70113-bib-0043] Zohner, C. M. , and S. S. Renner . 2017. “Innately Shorter Vegetation Periods in North American Species Explain Native‐Non‐Native Phenological Asymmetries.” Nature Ecology & Evolution 1: 1655–1660. 28963543 10.1038/s41559-017-0307-3

[gcb70113-bib-0044] Zohner, C. M. , A. Rockinger , and S. S. Renner . 2019. “Increased Autumn Productivity Permits Temperate Trees to Compensate for Spring Frost Damage.” New Phytologist 221: 789–795. 30240028 10.1111/nph.15445

