# Supplementary material for: Complex and Dynamic Effects of an Extreme Low Temperature Weather Event on Invasive Plant Populations and Resident Communities
Source: Glob Chang Biol. 2025 Mar 3;31(3):e70113. doi: 10.1111/gcb.70113 (PMC11874191; doi:10.1111/gcb.70113)
Supplement: Supplementary file 1 — Data S1. [file GCB-31-e70113-s001.docx]

**
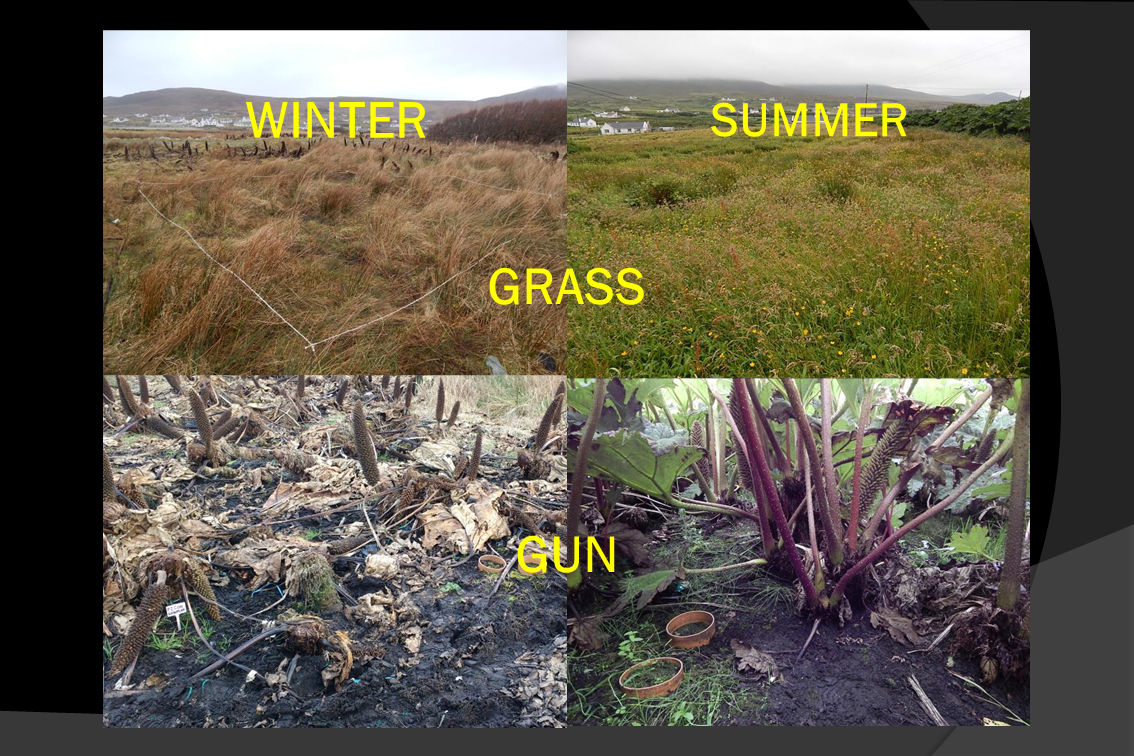

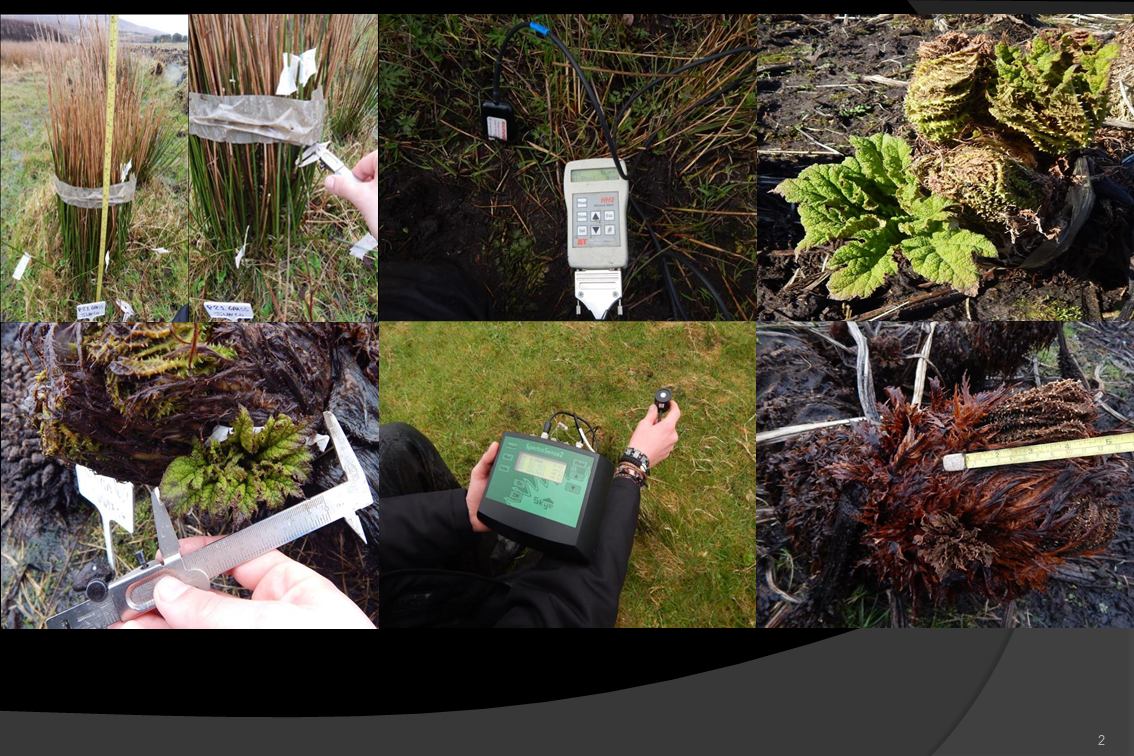
**

**Supplementary Figure 1 –** Pictures of the uninvaded semi-natural grassland plots dominated by *J. effusus* (GRASS) and areas invaded by *G. tinctoria* (GUN) in the winter (left) and summer (right), measurements of individuals of *J. effusus* and *G. tinctoria*, collection of environmental variables (soil temperature and moisture, and light levels), as well as the impact of the extreme weather event, called Storm Emma, on the apical growing points of the rhizome (healthy, in 2016, top, showing emerging leaves and lacking any shoots in 2018, after the EWE, bottom).

**
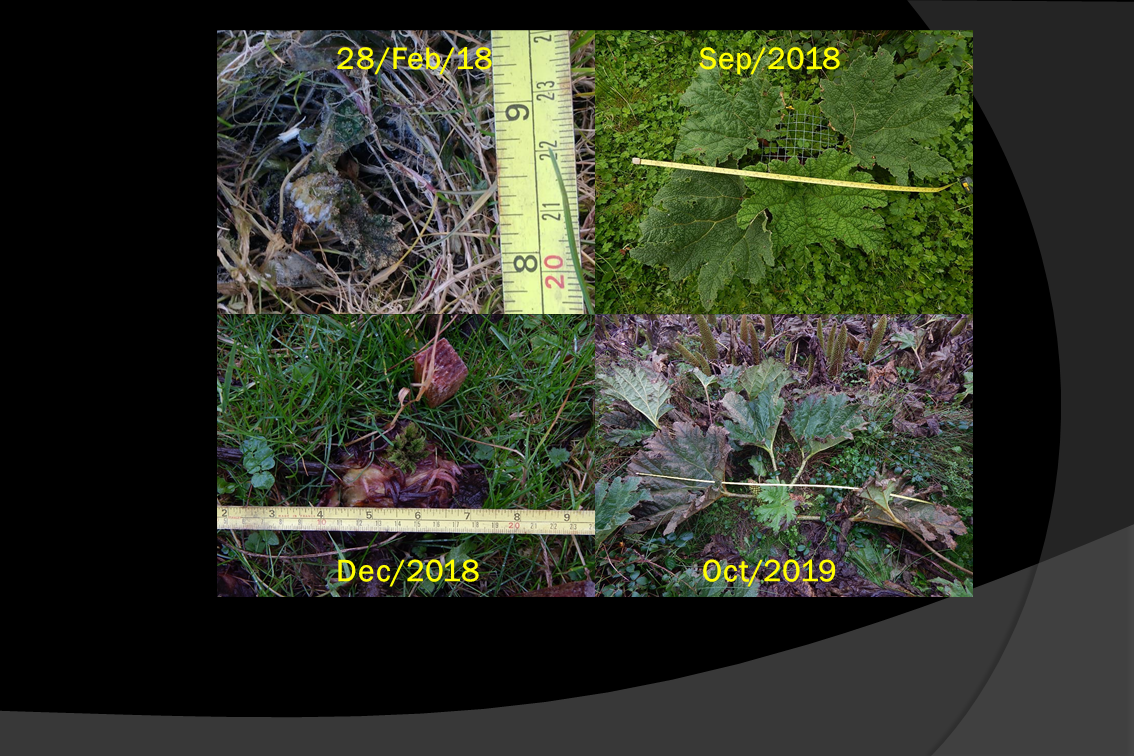
**

**Supplementary Figure 2 –** *Gunnera tinctoria* seedling developmental sequence showing the impact of low temperatures on the first day of the extreme weather event called Storm Emma (28/Feb/2018), six-months afterwards when seedlings reached their first peak in leaf area production (September/2018), initiation of a new leaf during winter (December/2018), and seedling with an inflorescence at the second peak of leaf area production (October/2019).





**Supplementary Figure 3 –** Seasonal variation in different growth-related parameters for *G. tinctoria* plants growing on Achill Island, Co. Mayo, Ireland from 2016-2019 (n = 5; mean ± 1SE). A = plant height (cm); B = *G. tinctoria* total petiole length (cm); and C = *G. tinctoria* total petiole thickness (cm). The vertical dotted line represents the occurrence of the extreme weather event called Storm Emma, in late February early March 2018.

**

**

**Supplementary Figure 4 –** Seasonal variation in (A) soil temperature and (B) moisture for uninvaded semi-natural grasslands (Grasslands) and areas invaded by *G. tinctoria* plants (*Gunnera*) on Achill Island, Co. Mayo, Ireland from 2016-2019 (n = 5; mean ± 1SE). The vertical dotted line represents the occurrence of the extreme weather event called Storm Emma, in late February early March 2018.

**Supplementary Table 1 –** List of species identified under uninvaded semi-natural grasslands (Grasslands) and invasive stands (*Gunnera*) at the end of the growing season (i.e., October) throughout 2016-2019, on Achill Island, Co. Mayo, Ireland.

| **Scientific Name** | **Family** | Grasslands | | | | *Gunnera* | | | |
| --- | --- | --- | --- | --- | --- | --- | --- | --- | --- |
|  |  | **2016** | **2017** | **2018** | **2019** | **2016** | **2017** | **2018** | **2019** |
| *Angelica sylvestris* L. | Apiaceae | X | X | X | X |  | X | X | X |
| *Apium nodiflorum* (L.) Lag. |  | X | X | X | X |  | X | X | X |
| *Berula erecta* (Huds.) Coville |  | X | X | X | X |  |  |  |  |
| *Cirsium palustre* (L.) Scop. | Asteraceae | X | X | X | X |  |  | X | X |
| *Cirsium vulgare* (Savi) Ten. |  | X | X | X | X |  | X | X | X |
| *Senecio jacobaea* L. |  |  |  | X | X |  |  | X | X |
| *Taraxacum officinale* F.H. Wigg. |  | X | X | X | X |  |  | X | X |
| *Myosotis secunda* A. Murray | Boraginaceae |  |  | X | X |  |  | X | X |
| *Cardamine pratensis* L. | Brassicaceae |  |  | X | X |  |  |  |  |
| *Cerastium fontanum* Baumg. | Caryophyllaceae | X | X | X | X |  |  |  |  |
| *Sagina procumbens* L. |  | X | X | X | X |  |  |  |  |
| *Dryopteris filix-mas* (L.) Schoott | Dryopteridaceae | X | X | X | X |  |  |  |  |
| *Equisetum arvense* L. | Equisetaceae | X | X | X | X | X | X | X | X |
| *Equisetum fluviatile* L. |  | X | X | X | X | X | X | X | X |
| *Trifolium pratense* L. | Fabaceae | X | X | X | X |  |  |  |  |
| *Trifolium repens* L. |  | X | X | X | X |  |  | X |  |
| *Gunnera tinctoria* Molina (Mirb.) | Gunneraceae |  | X | X | X | X | X | X | X |
| *Crocosmia x crocosmiiflora* (Lemoine) N.E. Br. | Iridaceae | X | X | X | X |  | X | X | X |
| *Juncus acutiflorus* Ehrh. | Juncaceae | X | X | X | X |  |  |  |  |
| *Juncus articulatus* L. |  | X | X | X | X |  |  | X |  |
| *Juncus effusus* L. |  | X | X | X | X | X | X | X | X |
| *Mentha aquatica* L. | Lamiaceae | X | X | X | X |  |  | X | X |
| *Prunella vulgaris* L. |  | X | X | X | X |  |  | X | X |
| *Lythrum salicaria* L. | Lythraceae | X | X | X | X |  |  | X | X |
| *Epilobium obscurum* Schreb | Onagraceae | X | X | X | X | X | X | X | X |
| *Callitriche stagnalis* Scop. | Plantaginaceae | X | X | X | X |  |  |  |  |
| *Plantago lanceolata* L. |  | X | X | X | X |  |  | X | X |
| *Veronica beccabunga* L. |  | X | X | X | X |  |  | X | X |
| *Agrostis capillaris* L. | Poaceae | X | X | X | X | X | X | X | X |
| *Agrostis stolonifera* L. |  | X | X | X | X | X | X | X | X |
| *Alopecurus pratensis* L. |  | X | X | X | X |  |  |  | X |
| *Anthoxanthum odoratum* L. |  |  |  | X | X |  |  |  |  |
| *Festuca ovina* L. |  | X | X | X | X |  |  | X |  |
| *Holcus lanatus* L. |  | X | X | X | X |  |  | X |  |
| *Lolium perenne* L. |  | X | X | X | X |  |  |  |  |
| *Paneion pratense* (L.) Lunell |  | X | X | X | X |  | X | X |  |
| *Poa annua* L. |  | X | X | X | X |  | X | X | X |
| *Poa trivialis* L. |  |  |  | X | X |  |  | X | X |
| *Persicaria maculosa* Gray | Polygonaceae | X | X | X | X |  | X | X | X |
| *Rumex acetosa* L. |  | X | X | X | X |  |  | X |  |
| *Rumex conglomeratus* L. |  | X | X | X | X |  | X | X | X |
| *Rumex crispus* L. |  |  |  | X | X |  |  | X | X |
| *Rumex obtusifolius* L. |  | X | X | X | X |  | X | X | X |
| *Anagallis tenella* (L.) L. | Primulaceae |  |  | X | X |  |  | X | X |
| *Ranunculus acris* L. | Ranunculaceae | X | X | X | X |  | X | X | X |
| *Ranunculus repens* L. |  | X | X | X | X | X | X | X | X |
|  |  |  |  |  |  |  |  |  |  |
| *Filipendula ulmaria* (L.) Maxim. | Rosaceae |  |  | X | X |  |  |  |  |
| *Potentilla anserina* L. |  | X | X | X | X |  |  | X | X |
| *Potentilla erecta* (L.) Raeusch. |  |  |  | X | X |  |  | X | X |
| *Potentilla reptans* L. |  |  |  | X | X |  |  | X | X |
| *Rubus fruticosus* L. |  | X | X | X | X |  | X | X | X |
| *Galium aparine* L. | Rubiaceae | X | X | X | X |  | X | X | X |
| *Galium palustre* L. |  | X | X | X | X |  |  | X |  |
| *Urtica dioica* L. | Urticaceae | X | X | X | X | X | X | X | X |
